# Simple topological task-based functional connectivity features predict longitudinal behavioral change of fluid reasoning in the RANN cohort

**DOI:** 10.1016/j.neuroimage.2023.120237

**Published:** 2023-06-19

**Authors:** Georgette Argiris, Yaakov Stern, Seonjoo Lee, Hyunnam Ryu, Christian Habeck

**Affiliations:** a Cognitive Neuroscience Division, Department of Neurology, Columbia University Irving Medical Center, 710 West 168th Street, 3rd floor, New York, NY 10032, United States; b Taub Institute, Columbia University, New York NY, United States; c Mental Health Data Science, New York State Psychiatric Institute, New York, NY, United States; d Department of Biostatistics, Mailman School of Public Health, New York, NY, United States; e Department of Psychiatry, Columbia University Irving Medical Center, New York, NY, United States

**Keywords:** Cognitive aging, RANN, TDA, System segregation, Task-based functional connectivity, Longitudinal

## Abstract

Recent attention has been given to topological data analysis (TDA), and more specifically persistent homology (PH), to identify the underlying shape of brain network connectivity beyond simple edge pairings by computing connective components across different connectivity thresholds (see Sizemore et al., 2019). In the present study, we applied PH to task-based functional connectivity, computing 0-dimension Betti (B_0_) curves and calculating the area under these curves (AUC); AUC indicates how quickly a single connected component is formed across correlation filtration thresholds, with lower values interpreted as potentially analogous to lower whole-brain system segregation (e.g., Gracia-Tabuenca et al., 2020). One hundred sixty-three participants from the Reference Ability Neural Network (RANN) longitudinal lifespan cohort (age 20–80 years) were tested in-scanner at baseline and five-year follow-up on a battery of tests comprising four domains of cognition (i.e., Stern et al., 2014). We tested for 1.) age-related change in the AUC of the B_0_ curve over time, 2.) the predictive utility of AUC in accounting for longitudinal change in behavioral performance and 3.) compared system segregation to the PH approach. Results demonstrated longitudinal age-related decreases in AUC for Fluid Reasoning, with these decreases predicting longitudinal declines in cognition, even after controlling for demographic and brain integrity factors; moreover, change in AUC partially mediated the effect of age on change in cognitive performance. System segregation also significantly decreased with age in three of the four cognitive domains but did not predict change in cognition. These results argue for greater application of TDA to the study of aging.

## 1. Introduction

Neuroimaging techniques have permitted the investigation of network dynamics in the brain, revealing synchronous patterns of neural activity between structurally-distinct but functionally-related regions (Sporns, 2013; Van Den Heuvel and Pol, 2010). To date, one of the main theoretical frameworks applied to the study of brain networks is graph theory (Rubinov and Sporns, 2010; Fornito et al., 2013). In this framework, a vertex (i.e., node) represents a specific brain region and an edge represents the link between pairs of vertices; this link is typically operationalized by Pearson’s correlation to capture the linear statistical dependency between temporal signals that are measured from anatomically distinct brain regions. Functional connectivity (FC) measured at rest has revealed that when these correlations are computed across multiple areas, the brain forms a complex large-scale network organization with modular architecture, where high segregation between modules (i.e., systems) supports healthy brain function (see Sporns and Betzel, 2016). However, the graph theoretic approach to network analysis comes with certain caveats. One potential limitation is that some metric calculations rely on network definitions contingent upon the parcellation scheme utilized; these schemes have spanned multiple approaches without formal consensus of connectome. Furthermore, analysis is often times limited to pairwise comparisons between edges, potentially over-looking broader dynamics across brain regions. Although other analytic techniques exist to circumvent this issue, such as network-based statistics or graph neural networks, the choice of analysis still largely comprises pairwise comparisons. Finally, graph-theoretical analyses have often adopted threshold selection to identify connections that are strong enough to constitute an edge in a binary graph (see Fornito et al., 2013) while more recent approaches have been adapted to allow for weighted and directional graphs. Considering network dynamics at different resolutions, network measures that rely on thresholding at a *single* correlation value to sparsify the network make strong a priori assumptions about the edge strength above which relevant information is carried and potentially restricts the rich network dynamics when attempting to capture individual- or group-level differences. To address these and other concerns, recent work has attempted to apply principles from algebraic topology to gain a better understanding of network dynamics without being constrained by arbitrary parameter selection and where the higher-order structure can be addressed beyond mere pairwise connections (Carlsson, 2020; Batteston et al., 2020).

Topological data analysis (TDA) is a tool for studying the underlying shape of high-dimensional data, beyond simple pairings of its constituent parts, in order to identify low-dimensional structures (Carlsson, 2009). Given the complex structure and graph-like behavior of brain connectivity, TDA applied to brain network analysis has gained traction in recent years (for a review of the method, see Caputi et al., 2021; Bullmore and Sporns, 2009). One prominent tool adopted in TDA towards the study of brain networks is persistent homology (PH; Lee et al., 2011). The PH approach allows for the computation of topological *features* of data that persist across different resolutions (Ghrist, 2008). These low-dimension topological features can be in different forms; for example, 0-dimension would represent connected components, 1-dimension would represent loops, and 2-dimension would represent voids. Data is considered to be a “point cloud” of *n* points (e.g., nodes scattered in space) where, in the case of 0-dimension, the number of connected components across these different resolutions, or filtration thresholds (e.g., edge density, correlation values, etc.) is calculated. As previously mentioned, PH is effective at circumventing threshold selection as features of the network are considered across multiple filtration thresholds (Caputi et al., 2021; Guerra et al., 2021; Lord et al., 2016; Petri et al., 2014) and has been utilized to quantify and visualize the evolution of brain networks (Stolz et al., 2021; Gracia-Tabuenca et al., 2020; Sizemore et al., 2018; Lee et al., 2017). The consideration of multiple filtration thresholds is different from sparsifying a matrix based on a single edge-weight threshold in that it captures how network configuration changes over all possible thresholds in a principled way. In the current paper, we consider 0-dimension connected components of task-based FC data, which translates to 0-dimensional simplicial complexes, and thus only considers the number of components formed between nodes. The number of connected components is tracked across each filtration threshold and refers to the Betti-0 number (B_0_), which is the number of sets of nodes connected by a sequence of edges (for an extensive review and TDA application to neuroscience, see Sizemore et al., 2019). To reiterate, in the simplest sense, the Betti-0 number at any given threshold is a measure of the number of connected components of the graph and thus captures the same information as the number of connected components. However, one major focus of PH is to capture the relevant topological features of a network over multiple scales or thresholding values. Prior work has utilized B_0_ curves, which are constructed from the B_0_ numbers that result from stepwise increases in filtration threshold, to study functional alterations between healthy controls and patient populations (Gracia-Tabuenca et al., 2020; Stolz et al., 2021). Plotting B_0_ against filtration values, Gracia-Tabuenca et al. (2020) calculated the area under the B_0_ curve (AUC) of resting-state FC, revealing attentional deficit hyperactivity disorder (ADHD)-related reductions in AUC. In principle, lower AUC should reflect overall fewer connected components as formation of the network into a single connected component requires fewer iterations across thresholds. One interpretation of lower values of AUC is that the brain may be engaged in less specialized information processing as interconnectivity is increased, and may thus reflect lower segregation, as the brain topology more quickly transitions to a single component.

In the present paper, we were interested in investigating age-related differences in the AUC of B_0_ when we apply PH to task-based FC. Research has shown that aging is typically linked to a decrease in segregation of functional brain systems (Varangis et al., 2019; Betzel et al., 2014), with this reduction linked to declines in cognition (Chan et al., 2021; Zonneveld et al., 2019; King et al., 2018; Chan et al., 2014). Additionally, a recent literature review of network measures that undergo changes across the lifespan utilized a certainty of evidence method to score reliability of findings across studies and found that age-related declines in functional segregation presents consistently and with high certainty. Therefore, we were also interested in comparing potential age-related differences in the AUC of B_0_ to functional network segregation in a longitudinal lifespan cohort. Longitudinal functional connectivity and behavioral data was collected from 163 participants tested in-scanner on four domains of cognition (i.e., reference abilities; RA) thought to comprise the breadth of age-related cognitive changes (Salthouse, 2009; Salthouse and Ferrer-Caja, 2003). A critical component to network neuroscience is understanding the complex link between network structure and behavior, with several methodologies having emerged to couple network analyses at the brain and behavioral levels (Blanken et al., 2021). Therefore, our primary goal was to investigate how age-related differences in AUC might relate to behavioral task performance. As reduced AUC of B_0_ has been observed in ADHD patients, potentially suggesting reduced segregation (Gracia-Tabuenca et al., 2020), we hypothesize that age should be linked with lower AUC and should relate to decreases in behavioral performance in some domains. TDA applications to network neuroscience is a recently burgeoning field, with emerging links between brain topology and behavioral features. Anderson et al. (2018) used PH to reveal a significant relationship between the temporal duration of topological features of dimension-0 and cognition in brain regions that are known substrates for these cognitive processes. However, we also wish to underscore the novelty of our study in that, while most studies have applied PH methods to resting-state FC, to the best of our knowledge, few have applied PH methods to task-based FC as is done here; moreover, we investigate longitudinal changes in network topology with the additional gain of relating this change to cognitive outcomes. Therefore, while our hypotheses are grounded in and guided by the existing literature, we refrain from forming strong a priori assumptions.

We also sought to compare longitudinal change in whole-brain system segregation to the PH approach, with particular focus on their utility in predicting cognitive change. In both analyses, we also considered a longitudinal measure of brain integrity (i.e., cortical thickness), which has been shown to decline throughout the lifespan (Fjell et al., 2015) and predict cognitive performance (Dominguez et al., 2021); cross-sectional work from our lab has also shown negative age associations with cortical thickness (CT) as well as revealing a complex relationship between CT and other demographic factors (Habeck et al., 2020). In addition, we considered total volume of white matter hyperintensities (WMH), which are lesions of presumed vascular origin that is a surrogate biomarker of cognitive decline (for a systematic review, see Debette et al., 2010; Deary et al., 2003; Cees De Groot et al., 2000). Prior cross-sectional work in our lab has shown significant correlations between WMH total volume and behavioral performance in two reference abilities, namely processing speed and memory (Moura et al., 2019). Here we consider longitudinal change in WMH total volume from baseline to follow-up.

## 2. Methods

### 2.1. Participants

One hundred sixty-three native English speaking, right-handed (Old-field Edinburgh Handedness Inventory; Oldfield, 1971) adults (age= 50.98 ± 16.53; range= 20 - 80 years), tested at two time points— baseline and 5-year follow-up— were included in the analysis. Participants were part of the Reference Ability Neural Network (RANN) cohort, which is a community-based cohort from the greater New York area. As we wished to maximize participant inclusion, we did not restrict our sample to participants who completed all 12 tasks of our design; each domain was treated separately and participants were required to have completed at least one task for each domain in a given testing session. The amount of data that is available may thus differ depending on domain and data type. For a list of participant demographics divided by decade of life, see Table 1a; for a list of the number of data points available for each domain, see Table 1b. Participants were recruited via random-market-mailing. All participants were screened for serious psychiatric or medical conditions, poor hearing and vision, and any other impediments that could hinder MRI acquisition; in addition, older participants were screened for dementia and mild cognitive impairment using the Dementia Rating Scale (DRS; Mattis, 1988) at both time points. All participants had less than 30% of their data “scrubbed,” as explained in Section 2.2.3. *fMRI Data Preprocessing*.

### 2.2. Procedure

FMRI data was acquired from participants as they performed 12 computerized cognitive tasks in scanner, each relating to one of four reference abilities (RA; Stern et al., 2014), at two time points (baseline and 5-year follow-up). At each testing time point, participants completed the battery of tasks over two sessions, each lasting for approximately 2 h and containing six of the 12 tasks belonging to two of the four RAs. The order of the two sessions was counterbalanced across participants, with tasks within each reference domain being presented in a fixed order. Prior to each scanning session, participants were familiarized with the six tasks relevant to the current session during an out-of-scanner training session, which was performed on a laptop computer. The mode of response for all but one task was keyboard button press; the picture-naming task used an oral response. Training sessions were self-paced, with breaks taken as needed, and participants were given the option of repeating the training session if desired. Assessment of task comprehension was made by a trained research assistant, with judgment based on participant’s subjective comfort with task completion. In a separate session, participants also completed a neuropsychological battery; however, results from this battery will not be addressed in the current paper.

#### 2.2.1. Stimulus presentation

Stimuli were back-projected onto an LCD monitor positioned at the end of the scanner bore. Participants viewed the screen via a tilted mirror system that was mounted on the head coil. When needed, vision was corrected-to-normal using MR compatible glasses (manufactured by SafeVision, LLC. Webster Groves, MO). Responses were made on a LUMItouch response system (Photon Control Company). E-Prime v2.08, operating on PC platform, was used for stimulus delivery and data collection. Task onset was electronically synchronized with the MRI acquisition device.

#### 2.2.2. Reference ability (RA) in-scanner tasks

Twelve cognitive tasks, each belonging to one of four reference domains, were presented in-scanner (for a complete description of task details, see Stern et al., 2014). For all tasks, with the exception of picture naming, responses were made via button press; picture naming, instead, required a vocal response. For episodic memory, fluid reasoning, and vocabulary domains, accuracy- measured as the proportion of correct trials to total trials included- was analyzed for each task. For the processing speed domain, RT data was analyzed for each task on accurate trials only. For the remainder of the document, an abbreviated version for each reference ability will be used: episodic memory– MEM, fluid reasoning– FLUID, processing speed– SPEED, and vocabulary– VOCAB. We also will interchangeably use the terms “domain” and “reference ability” to refer to our RAs. For the MEM domain, both study and test phases were scanned together and were not separated in the analysis. The tasks included were Logical Memory, Word Order Recognition, and Paired Associates. For the FLUID domain, the tasks included were Matrix Reasoning (adapted from Raven (1962)), Letter Sets (Ekstrom et al., 1976), and Paper Folding (Ekstrom et al., 1976). For the SPEED domain, the tasks included were Digit Symbol (adapted from Salthouse, 1998), Letter Comparison (Salthouse and Babcock, 1991), and Pattern Comparison (Salthouse and Babcock, 1991). For the VOCAB domain, the tasks included were Antonyms (Salthouse and Kersten, 1993), Picture Naming, and Synonyms (Salthouse and Kersten, 1993).

#### 2.2.3. fMRI data acquisition

Image acquisition was performed using a 3T Philips Achieva Magnet. Participants performed 12 fMRI tasks over the course of two, 2-hour MR imaging sessions; the same procedure was followed at both baseline and again at 5-year follow-up. At the onset of each session, a scout T1-weighted image was acquired to determine participant position. A T1-weighted MPRAGE scan was performed to capture participants’ brain structure, with the following parameters: TE/TR of 3/6.5 ms, flip angle of 8°, in-plane resolution of 256 × 256 voxels, field of view (FOV) of 25.4 × 25.4 cm, and 165–180 slices in the axial direction with a slice-thickness/gap of 1/0 mm. All scans used a 240 mm field of view. For the EPI acquisition, the following parameters were used: TE/TR of 20/2000 ms, flip angle of 72°, in-plane resolution of 112 × 112 voxels, and a slice thickness/gap of 3/0 mm. For FLAIR scan acquisition, the following parameters were used: 11,000 ms TR, 2800 ms TE, 256×189 voxels in-plane resolution, 23×17.96 FOV, and 30 slices with slice-thickness/gap of 4/0.5 mm. This sequence was utilized to quantify the white matter hyperintensities volumes. A neuroradiologist examined each participant’s scan for abnormality and any significant findings were reported to the participant’s primary care physician.

#### 2.2.4. fMRI data processing

Images were preprocessed using an in-house developed native space method (Razlighi et al., 2014). Briefly, the preprocessing pipeline first involved participant-level histogram computation for each volume for noise detection (Woolrich et al., 2001). Motion correction (MCFLIRT) was performed using the FSL package (Jenkinson et al., 2002), followed by slice-timing correction. All volumes were registered (6 df, 256 bins mutual information, and sinc interpolation) to the middle volume. Frame-wise displacement (FWD), as described in Power et al. (2012), was calculated from the six motion parameters and root mean square difference (RMSD) of the BOLD percentage signal in the consecutive volumes. To be conservative, the RMSD threshold was lowered to 0.3% from the suggested 0.5%. Contaminated volumes were then detected by the criteria FWD > 0.5 mm or RMSD > 0.3% and replaced with new volumes generated by linear interpolation of adjacent volumes. The sample means of the root mean square values of the detrended realignment estimates for characterizing motion artifact (Power et al., 2014) are reported in Table 1b. The percentage of new volumes generated (i.e., *scrubbing*) was tracked and considered as an inclusion criterion before regression analyses. Volume replacement was performed *before* temporal filtering (Carp, 2013). Flsmaths–bptf (Jenkinson et al., 2002) was used to pass motion-corrected signals through a bandpass filter with cut-off frequencies of 0.01 and 0.09 Hz. Finally, the processed data was residualized by regressing out the FWD, RMSD, left and right hemisphere white matter, and lateral ventricular signals (Birn et al., 2006). Task-unique effects were decidedly not regressed out of the functional connectivity time series because we wanted to ensure that removal of task-induced effects did not remove potential task-relevant information. While some studies have encouraged regressing out activations associated with task (e.g., Cole et al., 2019), other studies have suggested that task-induced changes may provide relevant information about task performance (see Greene et al., 2020). It has been suggested that removing task-related effects and computing correlations based on the ensuing residuals may be capturing more random fluctuations attributable to neither experimental design nor noise. Additionally, our analytic design relies on omnibus whole-brain analyses where no claims are being made as to specific task-related regions of activation, where region-to-region coactivations could arguably be meaningfully sensitive to stimulus effects, for instance. However, in light of the controversy surrounding potential task-induced effects that could meaningfully impact findings, we also created a second dataset where we regressed out the design matrix from the time series and performed subsequent analyses on the regressed time series. In anticipation of our findings, the main results were not significantly altered between differential treatment of the time series and the results from the task-regressed time series analysis, specifically for the Fluid Reasoning domain, is reported in the supplementary material section (see Supplementary Material Table ST3).

#### 2.2.5. Functional connectivity

T1 image segmentation was performed using FreeSurfer (Dale et al., 1999). The coordinates of 264 putative functional nodes, comprising 14 networks derived from a network partition scheme developed by Power et al. (2011), were transferred to each participant’s T1 space via non-linear registration of the participant’s structural scan to the MNI template using the ANTS software package. Next, a 10 mm diameter spherical mask, centered at each transferred coordinate, was generated, and intersected with the FreeSurfer gray matter mask in order to obtain the ROI mask for the 264 functional nodes. An intermodal, intra-subject, rigid-body registration of fMRI reference image and T1 scan was performed with FLIRT with 6 degrees of freedom, normalized mutual information as the cost function (Jenkinson and Smith, 2001), and used to transfer all ROI masks from T1 space to fMRI space. These transferred ROI masks were then used to average all the voxels within each mask to obtain a single fMRI time-series for each node. Pearson correlations were then performed for all pairwise combinations. This resulted in 264×263/2 = 34,716 fMRI connectivity pairs.

Given the differing nature of the task, the length of the time-series varied for each. The following represents the number of TRs (1 TR = 2000 ms) per task: MEM: Log_Mem- 210, Word_Order- 208, Pair_Assoc- 99; FLUID: Mat_Reason- 430, Letter_Sets- 430, Paper_Fold- 430; SPEED: Digit_Sym- 210, Letter_Comp- 195, Pattern_Comp- 190; VOCAB: Antonyms- 194, Pic_Name- 190, Synonyms- 194.

#### 2.2.6. White matter hyperintensities

FLAIR images were processed and white matter hyperintensities (WMH) segmented through a fully automatic supervised machine learning technique (Ithapu et al., 2014). In brief, this method utilizes random forest-based regression models to obtain a voxel-level class-specific labeling of the image. The final segmentation is a probability map between (0, 1), which denotes the likelihood that a given voxel is hyperintense, allowing for the calculation per subject of a normalized effective WMH volume. Additionally, periventricular and deep hyperintensity accumulations were separated using a ventricular template derived from the cerebral spinal fluid partial volume estimates to improve classification. White matter hyperintensity volume was defined as the sum of the labeled voxels multiplied by voxel dimensions. Each FLAIR sequence with a total WMH volume above 1000 mm^3^ was manually inspected to ensure that there were no visible discrepancies.

#### 2.2.7. Cortical thickness

Utilizing each participant’s T1-weighted MPRAGE image, cortical thickness measures were derived using the FreeSurfer (v5.1.0) software package (http://surfer.nmr.mgh.harvard.edu/). Although the estimation was automatically-generated, gray and white matter segmentation and spatial registration was manually checked based on the guidelines outlined in Fjell et al. (2009).

As we were dealing with longitudinal data from two time points, images were processed using the longitudinal stream (Reuter et al., 2012). Specifically, an unbiased within-subject template space and image is created using inverse consistent registration (Reuter et al., 2010). All processing steps are then initialized utilizing common information from the within-subject template, which has been shown to increase reliability and statistical power (Reuter et al., 2012). To obtain cortical thickness estimates, the gray/white matter boundary and cortical surface were first reconstructed (Dale et al., 1999). Next, at each point across the cortical mantle, measurements were calculated as the closest distance from the gray/white matter boundary to the gray matter. Mean cortical thickness was then derived across the entire cortical surface, yielding a single value.

### 2.3. Analytic approach

Topological data analytic measures were calculated using custom-written Python code (i.e., version 3) in conjunction with a TDA tutorial created by Centeno et al. (2022). Brain segregation and regression models were analyzed using custom-written MATLAB^®^ codes (Mathworks, Natick, Massachussets, USA). For post-hoc analysis, a measure of modularity Q (explained in 2.3.2. Network measures) was obtained using the Brain Connectivity Toolbox (Rubinov and Sporns, 2010).

#### 2.3.1. Regression analysis

##### 2.3.1.1. Linear regression analyses.

Demographic variables including Age, NART IQ (NART), Education (Edu), and Sex were included in every regression model. Cortical thickness and WMH total volume were also included as variables of interest. Variance attributed to mean scrubbing for each domain were removed from each brain measure prior to regression analysis. Model results are reported at the *p* < 0.05, uncorrected threshold, but significant findings are discussed for an FDR-corrected threshold of *q* = 0.05. FDR-correction was performed at the level of cognitive domain, or reference ability, where all models tested within a given domain were considered part of a “family of hypotheses”. For each domain, all model outcomes for both brain and behavioral measures as dependent variables were concatenated (=78 predictor p-values in total). FDR-correction was applied to this concatenated vector for each domain.

In order to standardize comparisons between tasks, behavioral scores at each time point were z-transformed using the mean and standard deviation calculated across all participants for each task separately at baseline. Given that speed tasks were measured as reaction time, z-score values were sign-inverted to correspond with accuracy scores, whereby higher scores always indicate better performance.

When treating regressions of longitudinal change, factors with measurements considered at both time points were residualized with respect to baseline values. That is, for WMH volumes, behavioral performance, cortical thickness, and Betti-0 number area under the curve (AUC) values (i.e., discussed in Section 2.3.2), the change values– calculated as follow-up (FU) minus baseline (BL) – were residualized with respect to baseline measurements. This was performed to account for baseline while preserving degrees of freedom in our models.

##### 2.3.1.2. Mediation analysis.

As a posthoc test, we also conducted mediation analysis for the Fluid Reasoning domain to examine a potential longitudinal mediating role of our TDA metric (i.e., change (Δ) in AUC of B_0_ between time points; described in section 2.3.2.1) for the relationship between age and change in cognition. We implemented a three-step bootstrapping process following the percentile method described by Preacher and Hayes (2004)- first, regressing ΔAUC on age to ensure a significant effect, after controlling for all demographic and brain factors; second, regressing Δcognition on age, while additionally controlling for the effect of ΔAUC; third, repeatedly simulating a comparison between these two regression models using a nonparametric bootstrapping of random samples (with replacement) approach to test the significance of the indirect effect of ΔAUC on the relationship between age and Δcognition. The results yielded point estimates for indirect, direct, and total effects in addition to the proportion of mediation, along with confidence intervals to ascertain significance. Bootstrapping was performed with 10,000 iterations. All regression models were adjusted for NART IQ, Education, Sex, ΔCT, and ΔWMH.

#### 2.3.2. Network measures

Participant-level adjacency (i.e., connectivity) matrices were constructed based on pairwise Pearson’s correlations between all 264 Power’s nodes; the direction of connectivity was not considered, thus matrices expressed undirected networks. FC correlation values (*r*) between nodes were converted to *Z*-coefficients using Fisher’s transformation (i.e., inverse hyperbolic tangent of *r*). In the case of TDA, matrices containing both negative and positive correlation values were utilized; however, a single network component was always formed before considering negative correlation as cutoff values. In the case of whole-brain system segregation, we utilized only the sparse matrix of positive edges, setting all negative edge weights to 0, as previously implemented (Chan et al., 2021). Thus, negative values for each brain measure were essentially treated commensurately. Both TDA and brain segregation measures were calculated at the participant-level, ultimately resulting in a single value per participant, per measure. As an additional measure to confirm the robustness of our eventual findings, we also calculated modularity Q, which is a method for community detection that measures the strength of partition of a network into modules, or non-overlapping communities of nodes (see Sporns and Betzel, 2016). For this calculation, again we utilized only the sparse matrix of positive edges.

##### 2.3.2.1. TDA.

**Fig. 1.**
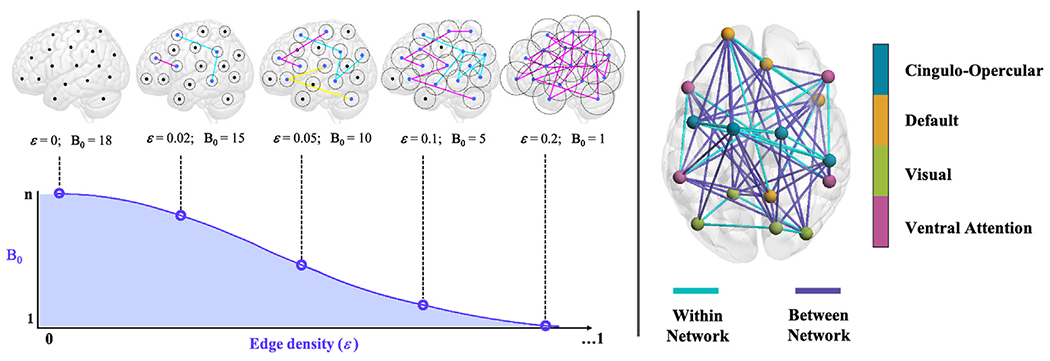
Schematics of functional connectivity measures. *Left panel: Schematic of Betti-0 (B*_*0*_*) curve.* Hypothetical Betti-0 curve for toy example of 18 nodes (n) in a brain network. Each of the five points indicated along the curve represents the Betti-0 number for a given filtration threshold (*ε*). As demonstrated, when *ε* =0, B_0_ is equal to the number of nodes in the network (=18), which each isolated node representing a component in itself. When *ε* =0.02, for example, where the edge weight cutoff value corresponds to 2% of the edge weight distribution, B_0_ is equal to 15, indicating 15 total connected components (13 isolated nodes and 2 *n*>1 connected components). When *ε* =0.2, all nodes are linked via at least one connection, forming a single component and thus yielding a B_0_ of 1. The B_0_ curve is characterized in terms of the area under the curve (AUC), shaded in purple, and is calculated as the trapezoidal integral across all filtration thresholds until a B_0_ of 1 is reached. Analysis is performed at the participant-level. *Right panel: Example of functional segregation*. Toy example of functional connectivity network with four networks and four nodes per network as defined by Power’s (2011) parcellation scheme. Turquoise lines represented edges that connect nodes belonging to the same (within) network and purple lines represent edges that connect nodes belonging to different (between) networks. Segregation is defined as the mean of all within-network connections minus the mean of all between-network connections, with this difference divided by the mean of all within-network connections. Segregation is computed for positive edge values only.

We used persistent homology to construct our graph filtrations and represent the network in topological space. As previously stated, we only considered 0-dimension homological features, which reflects the number of isolated nodes and connected components at different filtration thresholds (for a schematic example, see Fig. 1). Given the previously described instantiations of PH in the brain topology literature and desire to utilize the least derivative evaluation of connectivity for comparison to functional segregation, we decided to restrict our analyses to the 0-th homology as a first attempt at elucidating differences in network structure. We used a correlation threshold as the distance metric between nodes, similar to Rips filtration. The graph can be represented as a Rips complex, denoted by Rips(F, *ε*), where F represents a set of *n* points (i.e., nodes= 264) and *ε* the filtration value, which is a positive number that states if two nodes in F are connected; two nodes are considered linked if their distance is lower— i.e., correlation value *greater*— than *ε*. While different types of filtration thresholds can be utilized, such as 1-correlation (*r*) (e.g., Gracia-Tabuenca et al., 2020), here we use edge density to incrementally eliminate edges falling below a specific value as determined by percentage thresholding (e.g., Centeno et al., 2022). That is, rather than setting *ε* to 1-correlation (*r*), with fixed stepwise decreases in *r* in increments of 0.01, thresholding here was based on edge density, or an incremental increase of one percentile in the edge weight distribution to establish the cutoff weight (*W*). Each cutoff weight was essentially the participant-wise correlation value corresponding to each percentile in the distribution. That is, edges in the network were sorted from the highest weighted edge to the lowest weighted edge (i.e., shortest distance to the longest distance), a cutoff filtration distance of *W* is established based on the filtration threshold *ε* (e.g., *ε* = 1%, *ε* = 2%, *ε* = 3%, etc.), and edges between nodes remained for which the distance was shorter than the cutoff filtration distance, or *W*. We chose to use edge density in our sample to obviate potential age-related differences in the distribution of the connectivity edge weights.

The advantage to using edge density over a fixed correlation interval as a threshold is that potential participant-level differences in connectivity ranges are implicitly normalized.

As age-related differences in the variance of the correlation distributions may exist (e.g., Chen et al., 2017), we deemed edge density to be a more sensible approach for our sample. While our choice to obviate potential age confounders as practice was a priori motivated by conceivable shifts in the distribution based on the literature, our particular case here indeed showed age-related differences in variance for the Fluid Reasoning domain at both baseline and follow-up (BL: *r* = −0.25, *p* = 0.003; FU: *r* = −0.33, *p* = <0.001). However, in the effort of completeness, and again in anticipation of our findings, we also reran our analyses under Pearson correlation thresholding to ascertain the similarity of our findings between these two parameterizations specifically for the Fluid Reasoning domain; the table from the regression models are reported in the supplementary material section (see Supplementary Material Table ST4). The algebraic property extracted at each filtration threshold is the Betti-0 number (B_0_), which tracks the number of isolated nodes and those connected by a sequence of edges, which form components. At *ε* = 0, all nodes are connected components in and of themselves, in which case B_0_ is 264, or the sum of all nodes. At each iteration of filtration, B_0_ decreases until reaching a value of 1, where network connectivity has transition from all nodes being isolated to all being connected into a single component.

Similar to Gracia-Tabuenca et al., 2020, we computed the area under the curve (AUC) of B_0_ to capture this overall transition from isolated nodes to a single component, with smaller values of AUC suggesting that B_0_ decreases with smaller filtration values, reflects overall fewer connected components, and is arguably analogous to lower functional segregation. AUC was calculated using the trapezoidal integral method, which approximates the area of the region between two units, or as in this case, between two filtration thresholds. The integral over the entire B_0_ curve is achieved by summation across all areas between each filtration interval, from B_0_ = 264 (*ε* = 0) to the point at which B_0_ becomes 1.

##### 2.3.2.2. Whole-brain system segregation measure.

System segregation measures the extent to which a functional network is divided into distinct functional systems or subnetworks that are relatively independent of one another in terms of connectivity patterns (Wig, 2017; Chan et al., 2014). A prominent feature of functional brain network organization is the presence of subsets of areas that are highly interactive with one another while more sparsely interactive with other subsets of areas; this organization reflects distinct modules, or communities, of connectivity. Thus, in the context of specialized networks, modular brain network organization will be characterized by relatively high within-network relationships (e.g., high correlation) among brain regions and low between-network relationships among brain regions, reflecting functional specialization of information processing at the brain systems level (Chan et al., 2014). We used Power’s taxonomy (2011) to calculate system segregation as the difference between the mean within-network connectivity and the mean between-network connectivity, divided (i.e., normalized) by the mean within-network connectivity, defined by the following equation:

$$\text{Brain system segregation}=\frac{\frac{\overset{W}{\underset{w}{\sum }}\;Z_{w}}{W}-\frac{\overset{B}{\underset{b}{\sum }}\;Z_{b}}{B}}{\frac{\overset{W}{\underset{w}{\sum }}\;Z_{w}}{W}}$$

where *Z* is a Fisher’s z-transformed correlation value that represents an edge between a pair of nodes. *Z*_*w*_ represents the edges (correlations) between pairwise nodes that belong to within-network systems, *Z*_*b*_ represents the edges between pairwise nodes that belong to between-network systems, *W* is the total number of within-network edges across all subnetworks, and *B* is the total number of between-network edges across all subnetworks. This formula of brain system segregation is an updated version that more accurately specifies the precise computation (Chan et al., 2021). Higher brain system segregation values indicate greater distinction between functional systems. All 14 networks pertaining to Power’s (2011) parcellation scheme were utilized in calculating whole-brain system segregation.

##### 2.3.2.3. Modularity Q.

To foreshadow our findings, we also calculated a measure of modularity to which we compared the output from the TDA measure of AUC to confirm the robustness of our findings to another alternative metric. Modularity Q is a quality index that assesses the extent to which the correlation matrix can be partitioned into non-overlapping communities of nodes that maximize within-group connections and minimize between-group connections (Newman, 2006). A community structure of high quality, indexed by higher values of Q, is one where the partitioned communities are more internally dense than would be expected by chance (Sporns and Betzel, 2016). The calculation uses a deterministic modularity maximization algorithm to provide a single value of Q that quantifies the degree to which the network can be subdivided into clear, non-overlapping groups.

## 3. Results

**Fig. 2.**
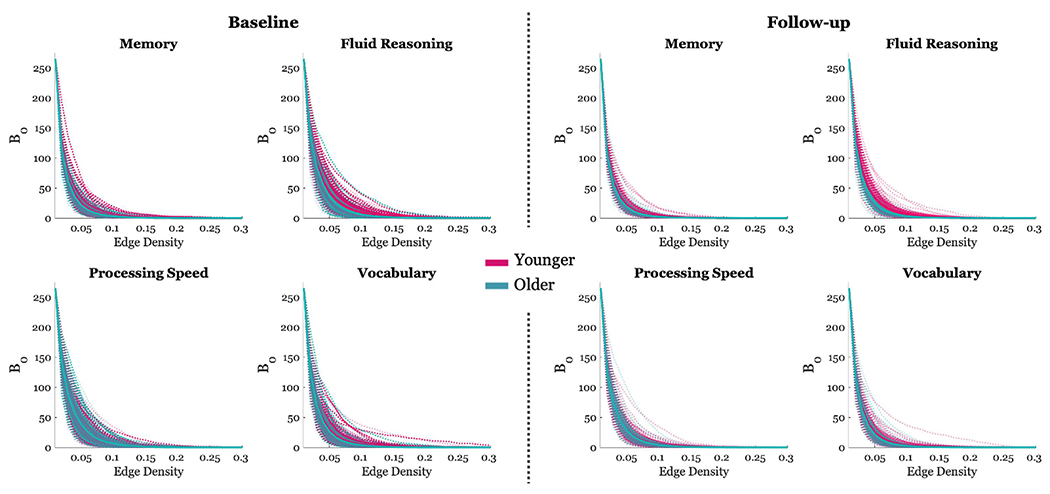
Line plots of B_0_ curves per participant at both baseline and follow-up. B_0_ values (y-axis) are plotted for edge density thresholds up to 0.3 (x-axis). Lines are colored based on participant’s age. Pink lines represent participants whose age is less than or equal to 50 years (i.e., younger) and green lines represent participants whose age is greater than 50 years (i.e., older). The bolded colored lines represent group means and the shaded bands represent one standard deviation around the mean.

For a list of all significant predictors (*p* < 0.05, *uncorrected* and corresponding FDR-adjusted p-values for *q* = 0.05) for each model, see Table 2. Full results for all model outcomes, irrespective of significance, are provided in Supplementary Data Table 1 (ST1). Results are reported for main findings at the FDR-corrected threshold. For a visualization of the B_0_ curves for each domain at both baseline and follow-up, see Fig. 2.

### 3.1. Functional connectivity models

#### 3.1.1. AUC models

Cross-sectional analyses of the AUC measure revealed a significant effect of Sex on AUC for the Memory domain at baseline (*β* = −0.223, 95% CI [70.8 −11.25], *p* = 0.035, $\eta_{\rho }^{2}=0.048$), with lower AUC for females. At follow-up, there was a non-significant positive trend, with a p-value of 0.088 after multiple comparisons correction, of Education on AUC, again for the Memory domain (*β* = 0.215, 95% CI [1.003 13.127], *p* = 0.088, $\eta_{\rho }^{2}=0.036$). Longitudinal analyses of AUC revealed a significant negative effect of Age on the change in AUC over time (*β* = −0.262, 95% CI [− 3.245 −0.591], *p* = 0.016, $\eta_{\rho }^{2}=0.06$), such that AUC disproportionately decreased over time with higher baseline age.

#### 3.1.2. System segregation models

Cross-sectional analyses of system segregation revealed significant negative effects of Age on all domains, at both baseline and follow-up (*p* <=0.038 for all models; see Table 2 for model parameters). Longitudinal analyses of system segregation revealed a significant negative effect of Age on Memory (*β* = −0.235, 95% CI [−0.002 −0.0003], *p* = 0.046, $\eta_{\rho }^{2}=0.046$), Fluid Reasoning (*β* = −0.272, 95% CI [−0.003 –0.0006], *p* = 0.009, $\eta_{\rho }^{2}=0.063$), and Processing Speed domains (*β* = −0.293, 95% CI [−0.002 −0.0005], *p* = 0.012, $\eta_{\rho }^{2}=0.075$). For a plot of the relationship between Age and change in system segregation after adjusting for other demographic and brain integrity factors, see Fig. 4.

#### 3.1.3. Modularity Q

As a posthoc analysis, we focused on longitudinal changes in modularity Q. Longitudinal analyses revealed a non-significant effect with p-value of 0.05<*p*<0.1 after multiple comparisons correction for the Fluid Reasoning domain, with a negative trend between Age and change in modularity Q (see Supplementary Material SF1 for a scatterplot).

### 3.2. Behavior models

We report the results from the behavioral performance regression analyses when the AUC of B_0_ measure is included as predictor. More specifically, we focus on findings from the longitudinal analyses with AUC as predictor. As a fundamental aim of our paper was to compare connectivity measures and their utility in predicting behavior, we also created models with functional segregation as predictor, which, apart from the connectivity measure itself, yielded similar results for the effects of demographics and other measures of brain integrity on behavior. For this reason, results for behavioral models are only reported once in both Table 2 and supplementary Table 1.

#### 3.2.1. Behavioral performance regressions

Longitudinal analyses with behavioral performance as outcome revealed a significant negative effect of baseline Age on change in behavioral performance over the 5-year span for the domains of Memory (*β* = - 0.24, 95% CI [−0.016 −0.002]*p* = 0.037, $\eta_{\rho }^{2}=0.05$), Fluid Reasoning (*β* = −0.415, 95% CI [−0.022 −0.009], *p* < 0.001, $\eta_{\rho }^{2}=0.163$), and Processing Speed (*β* = − 0.356, 95% CI [−0.019 −0.006], *p* = 0.0014, $\eta_{\rho }^{2}=0.108$). There was also a significant positive effect of baseline NART on change in behavioral performance for the domains of Memory (*β* = 0.535, 95% CI [0.024 0.055], *p* < 0.001, $\eta_{\rho }^{2}=0.165$), Fluid Reasoning (*β* = 0.428, 95% CI [0.017 0.044], *p* < 0.001, $\eta_{\rho }^{2}=0.142$), and Vocabulary (*β* = 0.456, 95% CI [0.017 0.047], *p* < 0.001, $\eta_{\rho }^{2}=0.139$). There were also two non-significant effects with p-values of 0.05<*p*<0.1 after multiple comparisons correction: a positive trend of change in CT on change in behavioral performance for the Memory domain, with lower cortical thickness over time associated with reduced AUC and a negative trend of change in WMH on change in behavioral performance for the Fluid domain, such that increases in white matter hyperintensities over time were linked to declines in performance. Notably, there was also a significant positive effect of the change in AUC on change in behavioral performance for the Fluid domain (*β* = 0.277, 95% CI [0.0006 0.002], *p* = 0.003, $\eta_{\rho }^{2}=0.094$), with higher AUC linked to better performance over time. When functional segregation was used as a predictor in the behavior models, there was no significant effect in any domain, neither with FDR-correction of *q* = 0.05 nor uncorrected *p* < 0.05.

#### 3.2.2. Posthoc analyses of the fluid reasoning domain

To confirm the validity of our finding demonstrating a positive relationship between ΔAUC and ΔCognition, we also performed several posthoc analyses. First, permutation testing was utilized in order to establish a null distribution to which we could compare our statistic calculated from the regression analysis. We achieved this by randomly shuffling predictor variables across subjects, maintaining within-subject predictor assignment across the independent variables but disrupting their corresponding assignment to the behavioral performance dependent outcome. This process was repeated 10,000 times. Significance was assessed as the ratio between the number of times the value of the null distribution generated a difference greater than the one observed in the data divided by the number of permutations (=10,000). The effect of ΔAUC on ΔCognition for Fluid Reasoning was confirmed to be significant at *p* < 0.001. Next, we conducted split sample analysis where the functional time series was divided into two parts and the regression analyses were repeated in order to ensure that the findings are relevant at shorter time scales. Longitudinal behavioral regression analyses for each of the two truncated times series still revealed a significant positive effect of ΔAUC on ΔCognition (*First: β* = 0.238, 95% CI [0.0005 0.002], *p* = 0.004, $\eta_{\rho }^{2}=0.064$; *Second: β* = 0.227, 95% CI [0.0004 0.002], *p* = 0.004, $\eta_{\rho }^{2}=0.065$). We then looked at the relationship between modularity Q, a potentially analogous measure to system segregation that is additionally agnostic to network parcellation and change in cognition. When modularity Q was used as a predictor in the behavior models, there was no significant effect in the Fluid or any other domain, neither with FDR-correction of *q* = 0.05 nor uncorrected *p* < 0.05.

As a final posthoc test, we also compared models with all combinations of predictors (=127 model combinations in total) for the Fluid Reasoning domain and compared BIC values to select the most parsimonious model that best predicted longitudinal change in cognition. The winning model contained Age, NART IQ, ΔAUC, and ΔWMH burden as predictors (*F*(4, 127) = 16.56, *p* < 0.001, *f^2^* = 0.475, with an adjusted R^2^ of 0.322.

#### 3.2.3. Mediation model

**Fig. 3.**
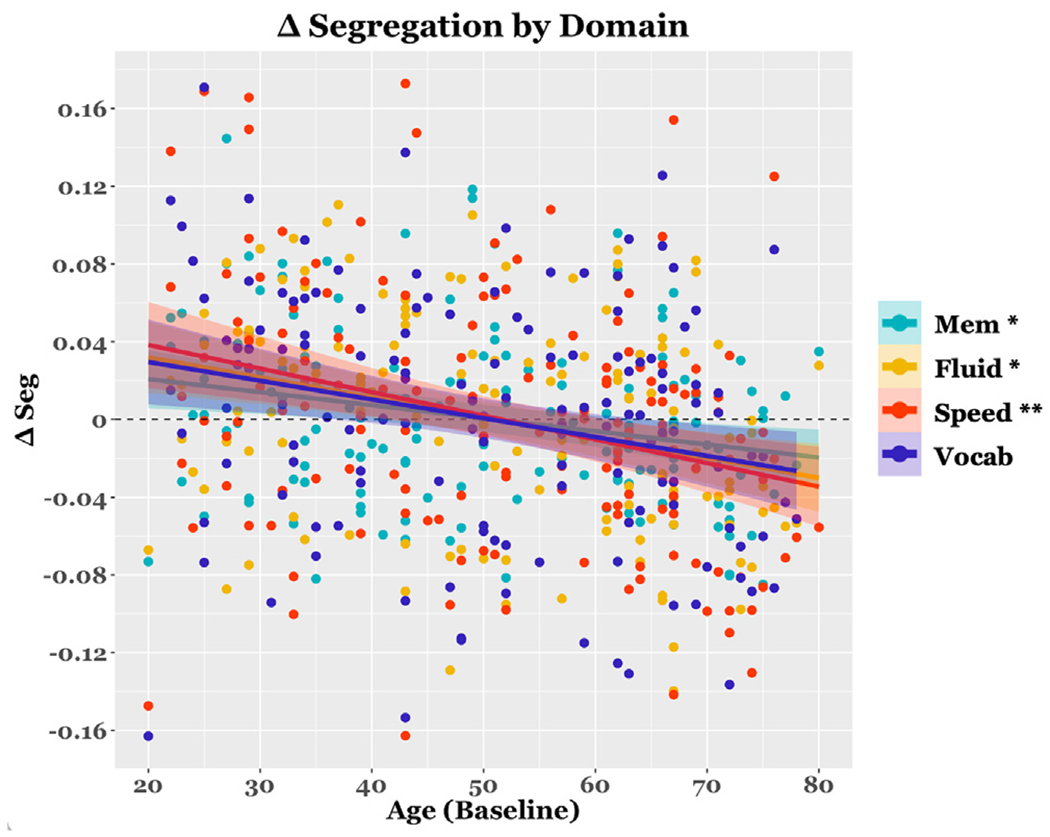
Scatterplot depicting the relationship between baseline Age and longitudinal change in system segregation. Colored dots represent each participant’s segregation value per domain.

**Fig. 4.**
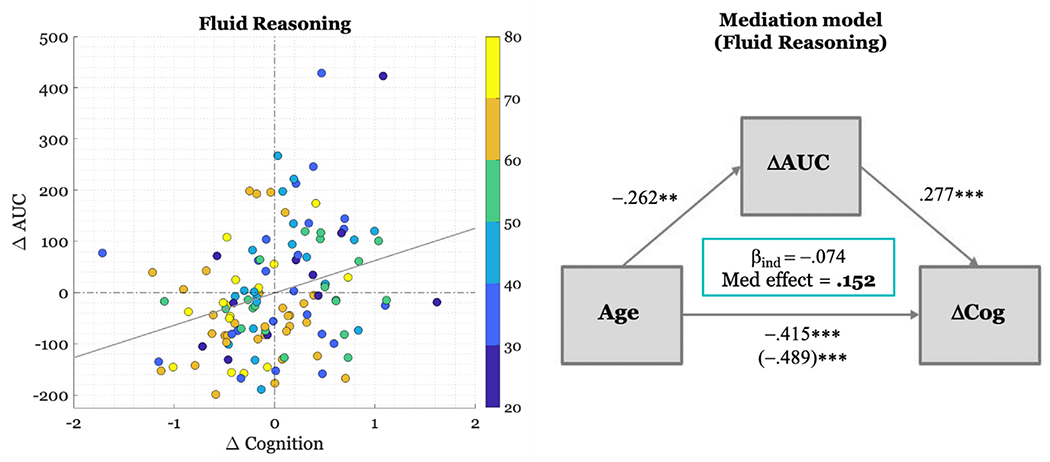
*Depiction of relationship between baseline Age, longitudinal change in AUC (*Δ*AUC), and longitudinal change in cognition (*Δ*Cognition) for Fluid Reasoning.* *Left panel:* Scatterplot where each dot represents participant’s ΔAUC and ΔCognition value. The color of the dot depicts the age band to which the participant belongs. As a reminder, behavioral performance was standardized using baseline mean and standard deviation across all participants. ΔACognition values have been adjusted for baseline behavior, NART, Education, Sex, ΔCT, and ΔWMH and thus represent the raw residuals after this adjustment. ΔAUC values have been adjusted for baseline AUC. The gray line represents the least-squares fit (*β* = 0.245). *Right panel:* Schematic of mediation model illustrating model parameters. Values along the arrows represent standardized beta coefficients for the effect of Age on ΔAUC, the effect of ΔAUC on ΔCognition (i.e., ΔCog) after adjusting for Age, and the direct effect of Age on ΔCog. The total effect of Age on ΔCog is represented in parentheses. The beta coefficient for the indirect mediating effect of ΔAUC (i.e., *β*_ind_) in addition to the proportion of the effect that is mediated is reported in the green box.

Given the effect of Age on ΔAUC and the effect of both Age and ΔAUC on ΔCognition for the Fluid Reasoning domain, we also performed a posthoc mediation analysis to test whether ΔAUC mediated the effect of Age and ΔCognition. NART, Sex, Education, ΔCT, and ΔWMH were included as covariates in the models. We observed a significant mediation (indirect) effect of ΔAUC (Average Causal Mediation Effects (ACME) = −0.074, 95% CI = [−0.0057 –0.0006], *p* = 0.007) with a significant direct effect of Age on ΔCognition, indicating a negative partial mediation of ΔAUC on the effect of Age on Δcognition. This finding indicates that advancing age disproportionately leads to a reduction in AUC across time, which, in turn, leads to a greater reduction in behavioral performance in Fluid Reasoning. The proportion of mediation relative to the total effect was 0.152 (C1 = [0.037, 0.32], *p* = 0.007). Fig. 3 provides a scatterplot depicting the relationship between Age, ΔAUC, and ΔCognition in addition to the mediation model parameters.

Colored error ribbons represent the 95% confidence band. Change in segregation (i.e., ΔSeg) values have been adjusted for baseline segregation, NART, Education, Sex, ΔCT, and ΔWMH and thus represent the raw residuals after this adjustment. Asterisks indicate statistical significance at threshold levels *p*<0.05 (*), *p*<0.01 (* *), and *p*<0.001(* * *).

## 4. Discussion

In the present study, we applied PH, a tool from topological data analysis (TDA), to the study of longitudinal change in task-based functional connectivity (FC) network dynamics across the lifespan. We sought to compare results from TDA analyses to those of a whole-brain graph theoretical measure (i.e., brain system segregation), which has been shown in prior studies to decline with age (Chan et al., 2021; Varangis et al., 2019; Betzel et al., 2014). Importantly, we were interested in comparing the utility of each measure in predicting longitudinal change in cognitive performance. We found that, while system segregation showed consistent declines with age in nearly all domains, it did not predict behavior at any time point, neither cross-sectionally nor longitudinally. An additional measure, modularity Q, was considered in posthoc analysis and also did not predict behavior, neither cross-sectionally nor longitudinally. Conversely, the TDA method, which resulted in calculating the AUC of Betti-0 curves across multiple filtration thresholds, predicted longitudinal change in behavior in the Fluid Reasoning domain. This finding withstood permutation testing and split-sample analysis of the time series data, providing a compelling case for its robustness.

Relatively few studies to date have applied TDA methods to network analyses of brain connectivity, though it has gained momentum as a promising new tool for the study of brain dynamics. Given the intrinsic structure of its constituent parts and potentially disordered dynamics, brain connectivity can suitably be considered a complex system to which the study of complex patterns can be and has been increasingly applied (Caputi et al., 2021; Bullmore and Sporns, 2009). In a seminal work by Lee and colleagues, PH was applied to brain connectivity using Pearson’s correlation thresholding to generate Betti-0 barcodes (i.e., the “lifespan” or “persistence” of topological features, or components, across filtration thresholds) at the group level to distinguish between patient groups (i.e., autism spectrum disorder (ASD) or ADHD) and cognitive healthy controls. Other more recent work has extended these methods to include other low-dimension topological features (e.g., Betti-1 numbers) in addition to support vector machine classification to distinguish between patient and control groups (Stolz et al., 2021). Individual-level applications of PH methods though have been limited. Gracia-Tabuenca et al. (2020) used Pearson’s correlation as a filtration threshold to generate individual-level Betti-0 curves, which were subsequently utilized to calculate the area under these curves (i.e., AUC). Their results revealed significant differences between ADHD and cognitively healthy individuals, with ADHD individuals forming a single connected component more quickly than healthy controls, as indexed by lower AUC values. This finding has been interpreted as being analogous to lower system segregation, given that a lower AUC should, in principle, reflect fewer filtration thresholds to arrive at a single connected component. Lower system segregation has typically been associated with negative functional and cognitive outcomes, particularly with respect to aging as well as pathological conditions (Xu et al., 2021; Ewers et al., 2021; Chan et al., 2021; Varangis et al., 2019; Chong et al., 2019; Chan et al., 2014).

In the present analysis, we also grounded our hypothesis in this assumption of segregation, and indeed, did show that both system segregation and AUC of the Betti-0 curve displayed longitudinal declines with age for the Fluid Reasoning domain. A posthoc consideration of modularity Q as an analogous measure of segregation of the system indicated a similar negative trend with Age, although it did not survive multiple comparisons correction. However, we do not wish to make strong interpretative claims as to the definitive meaning of what AUC represents, as empirical evidence for bivariate assumptions is lacking. First, we wish to highlight that the calculation of system segregation depends on a specific network configuration, whereas the calculation of AUC is agnostic to the neural network to which a node belongs. Modularity Q, whose calculation was also agnostic to parcellation, showed a similar trend of results, albeit non-significant after correction. Second, Lee et al. (2011) showed shorter barcodes of B_0_ charting the persistence of isolated components at different correlation filtration thresholds for the control group compared to either the ASD or ADHD group, indicating that brain connectivity merged *faster* into a single component for the control group. They attributed these findings to both general under-connectivity and increased local overconnectivity in the ASD and ADHD groups. It is important to reiterate that we calculated AUC for individual B_0_ curves, similar to Gracia-Tabuenca et al. (2020), and additionally used edge density as a filtration threshold, leaving open the possibility for alternative interpretations. One such alternative is that AUC is a measure of network *integration* and not *segregation*, where the assimilation of information is aided by network “hubs” that ensure efficient and rapid communication between segregated, spatially distributed network communities (for a review, see Deco et al., 2015). However, filtration thresholds here consider “distance” only in terms of correlation and is agnostic to both coordinate space as well as network affiliation; thus, strict translation from the PH method adopted here to existing measures of brain connectivity is limited. However, recent work in our lab has explored the mathematical properties of the Betti-0 curve and showed that the AUC may reflect backbone structure of information flow that persists across filtration thresholds; this structure may actually describe brain-integrating processing, as opposed to brain segregation (Ryu et al., 2023).

Finally, it is worthy to note that neither high segregation nor high integration are necessarily beneficial in an absolute sense, but that successful information processing relies on the balance between independent processing in specialized subsystems in addition to global cooperation between those subsystems (Wang et al., 2021).

Nonetheless, we also found a significant relationship between longitudinal reductions in AUC and longitudinal decline in cognitive performance again for the domain of Fluid Reasoning, indicating that higher AUC, as measured by edge density filtration thresholds, is linked to better performance over time. The robustness of this finding was evidenced by it having withstood both permutation testing and split sample analysis of the time series. To our knowledge, very few studies have analyzed the effect of PH measures on behavioral performance. One study by Anderson et al. (2018) applied PH barcode analysis to resting state FC in both the time and space domains to investigate its relationship to several types of cognition and personality. Notably, they found that Fluid Intelligence was negatively correlated with persistence barcode values in both the time and space domains. Moreover, of all 17 types of cognition or personality considered, Fluid Intelligence displayed the most consistent relationship to persistence barcode values. It is worth noting though that the interpretation of longer persistence barcodes depends on the specific topological features that the barcodes represent. In the case of connected components and the length of a node’s participation in a specific topological feature, longer barcodes could indicate a greater degree of integration in the graph. However, if the node is an isolated component that persists as such as the filtration parameter is varied, it could be interpreted as indicating higher segregation in the graph. While is difficult to draw definitive comparisons between Anderson et al. (2018) results and the present findings, the fact that the relationship between barcode values and both Age and Fluid Reasoning performance were consistently in the opposite direction to our findings might suggest that AUC and persistence barcode values might be capturing different aspects of the topological organization. In the spatial domain, the brain regions displaying significant correlation between barcode values and Fluid Intelligence were located in the association cortex, particularly across frontoparietal attentional regions. These findings are similar to what would be expected from a parieto-frontal integration theory of intelligence, which asserts that the parietal and frontal cortices comprise the core regions involved in intelligence (Jung et al., 2007). However, it is important to underline the distributed nature of the networks underlying intelligence (Colom et al., 2010), given its definition as the capacity to think logically and solve problems in novel situations, independent of acquired knowledge (Cattell, 1987). As a general mental ability, it has been associated with a plethora of structural and functional brain properties, including cortical thickness in prefrontal, temporal and association cortical areas, white matter integrity of long association fibers (see Deary et al., 2010 for a comprehensive review of biological factors), and strength of resting-state FC between brain regions distributed in frontal, parietal, occipital, and limbic lobes (Song et al., 2008). Areas displaying significant correlation between intelligence and FC in the study by Song et al. (2008) supported an exploratory network whose activation “efficiency” was modulated based on intelligence level. Even when considering network dynamics alone, it is perhaps unsurprising that Fluid Reasoning should be a domain sensitive and suitable to being explored using TDA measures, particularly when utilizing whole brain connectomics.

It is important though to note that prior studies mentioned here have investigated the relationship between resting-state FC, not task-based FC, and intelligence. While it has been shown that intelligence is related to less reconfiguration and thus greater similarity between resting-state and task-based networks (Schultz and Cole, 2016), there is not necessarily a one-to-one mapping of the neural substrates involved. Beyond providing a potentially more accurate neural representation of the mental states engaged during task performance, it has been suggested that task-based FC is a better predictor of independent fMRI activations across several task conditions, with task-related changes to FC playing an important role in dynamically reshaping brain network organization (Cole et al., 2021). Thus, investigating the neural substrates of functional engagement during task performance can offer critical insight into the link between brain and cognitive processes. In the current study, we showed that our PH measure of AUC significantly predicted cognitive performance in Fluid Reasoning during task-based FC; moreover, we demonstrated this relationship longitudinally. To the best of our knowledge, our study is the first to provide longitudinal evidence for the utility of PH in predicting cognitive performance. Notably, we also considered demographic and brain integrity factors in our models, with AUC predicting cognition above and beyond variables such as cortical thickness and WMH total volume.

In addition to demonstrating a significant relationship between longitudinal change in AUC and cognitive performance in Fluid Reasoning, we also showed that longitudinal change in AUC mediated the effect of age on longitudinal change in cognitive performance. Previous work in our lab has shown significant longitudinal decline in Fluid Reasoning, with older age associated with steeper decline (Gazes et al., 2021). Here, we provide one mechanism through which advancing age might lead to increased cognitive decline. When we compared linear regression models that would optimally predict longitudinal change in cognition for the Fluid Reasoning domain, we found that longitudinal change in cognition was best predicted by age, change in AUC, NART, and white matter hyperintensity burden. NART IQ, which has been utilized as a proxy for cognitive reserve, or individual differences in the cognitive or neural processes that act as coping mechanisms against compromised brain function (Stern, 2009), was significantly positively related to longitudinal change in cognitive performance. Conversely, an increase in WMH total volume was negatively related to cognitive performance. Previous work in our lab has demonstrated cross-sectional relationships between WMH total volume and performance on the Memory and Processing Speed domains; only regional measures of WMH showed inverse correlations between WMH and cognitive performances across all four cognitive domains. A recent longitudinal study by Wang et al. (2020) analyzed ADNI data from eight cognitive domains and showed that bigger WMH volumes were correlated with worse performance on several abilities such as memory and executive function, in addition to contributing to AD conversion.

A curious finding from our study is that system segregation did not predict cognition in any of the four domains. While this highlights the advantage of the PH method for the Fluid Reasoning domain, it also stands at odds with other studies that have found links between system segregation and age-related differences in cognitive performance (Zonneveld et al., 2019; King et al., 2018; Chan et al., 2014). One reason for the absence of such an effect could be that past studies investigated FC at rest and not during task engagement. Cross-sectional work from our own lab though has shown that the relationship between in-scanner behavioral performance and task-based network connectivity heavily depends on the cognitive domain (Varangis et al., 2021). Furthermore, the most robust age effects on network connectivity appeared for the Fluid Reasoning domain. Findings from the present study showed significant age-related longitudinal decline in system segregation in the Memory, Fluid Reasoning, and Speed domains. These results corroborate and extend previous findings that have displayed cross-sectional evidence for age-related reductions in system segregation.

The present study, however, is not without limitations. First, given the more recent application of TDA to the study of functional brain networks, it is difficult to provide strong definitive claims as to what aspect of connectivity the measure B_0_ - AUC is precisely capturing. Theoretically, two vastly different B_0_ curves can yield the same value of AUC. While we did not systematically address this potential outcome, we plotted participant-level B _0_ curves to detect anomaly, instead observing an arguable homogeneity of shape. However, one way to address this caveat would be to consider other aspects of the B_0_ curve (e.g., inflection points, higher-order moments of the curve distribution) in addition to other filtration methods. In the current study, we utilized edge density as our filtration threshold, which is one way of dealing with differences in correlational variation across individuals. This approach naturally allows for the absolute correlation value at each filtration threshold to differ across individuals. Instead, decreasing the correlation cutoff by the same increment across individuals (e.g., 1-r) could yield different results. How to treat and interpret the use of different filtration thresholds is one launching point for future work. Next, as previously mentioned, our method is agnostic to both coordinate space as well as nodal network affiliation. One future direction would be to try to incorporate other types of network information into PH-based measures to gain a more precise understanding of regional contributions to the underlying shape of the network. It could be that specific networks may contribute more or less to formation of a single component; this could also be the case for system segregation, where some studies have divided between association and sensorimotor systems, for instance (e.g., Varangis et al., 2021). Another analysis currently underway in our lab is investigating PH measures in resting-state, which would shed light on the generalizability of the current findings to other functional connectivity states. Furthermore, calculation of Betti-0 numbers, by definition, occurs until a single component is formed (i.e., all nodes are connected to at least one other node without recursion). However, network configuration beyond the establishment of a single component is not considered. As we only considered 0-dimensional features of topological space, it might behoove to consider other low-dimension topological features, such as other simplices (i.e., loops and voids) which might confer a more detailed understanding of the shape of the network.

## Supplementary Material

supplementary material

## Figures and Tables

**Table 1a:** Participant demographics divided by decade of life.

Age Bracket	N	Sex		Age		NART		Education	
		Male	Female	Mean	SD	Mean	SD	Mean	SD
20–29 years	22	8	14	25.68	2.7	113.43	7.99	15.27	1.96
30–39 years	27	11	16	34.8	2.94	111.99	7.61	16.78	2.78
40–49 years	24	14	10	44.58	2.7	117.11	8.71	16	2.57
50–59 years	25	10	15	54.2	3.24	120.24	6.23	16.2	1.96
60–69 years	42	24	18	64.76	2.58	118.74	7.83	16	2.43
70–80 years	23	11	12	73.74	2.49	121.62	6.23	17.7	2.48

**Table 1b:** Available data for each domain in addition to root mean square (RMS) estimates of motion artifact.

	Mem	Fluid	Speed							Vocab		
	BL	FU	D	BL	FU	D	BL	FU	D	BL	FU	D
fMRI	159	157	154	159	153	150	162	158	157	160	152	150
Behavior	157	156	150	159	159	155	155	149	141	153	153	143
fMRI RMS	.375	.428	.08	.553	.67	.12	.381	.518	.136	.511	.544	.042

Total number of data points (fMRI scans or behavioral performance scores) available per domain. In addition, we report the mean RMS values denoting motion artifact.

**Table 2:** List of significant predictors (*p* < 0.05, uncorrected) and accompanying FDR-corrected p-values for all linear regression models.

Predictor	Time	Domain	Predictor	*β*	p	FDR-p	CI	$\eta_{\rho }^{2}$
AUC	BL	Mem	Sex	−0.223	.007	.035*	[−70.8 −11.25]	.048
	FU	Mem	Edu	.215	.023	.088†	[1.003 13.127]	.036
		Fluid	Sex	−0.178	.032	.104	[−76.91 −3.501]	.033
		Fluid	Edu	.189	.044	.122	[.24 17.141]	.029
	FU-BL	Fluid	Age	−0.262	.004	.016*	[−3.245 −0.591]	.06
		Fluid	Edu	.21	.037	.112	[.589 18.76]	.03
SEG	BL	Mem	Age	−727.	<0.001	<0.001***	[−0.003 −0.002]	.19
		Mem	WMH	.232	.032	.112	[.0009 0.019]	.03
		Mem	Sex	.149	.045	.146	[.0004 0.032]	.027
		Fluid	Age	−0.623	<0.001	<0.001***	[−0.004 −0.002]	.14
		Fluid	WMH	.225	.041	.119	[.0005 0.023]	.028
		Speed	Age	−0.662	<0.001	<0.001***	[−0.004 −0.002]	.18
		Vocab	Age	−0.575	<0.001	<0.001***	[−0.003 −0.001]	.127
	FU	Mem	Age	−0.37	.004	.021*	[−0.002 −0.0004]	.057
		Fluid	Age	−0.39	.002	.01**	[−0.003 −0.0006]	.067
		Speed	Age	−0.532	<0.001	<0.001***	[−0.0004 −0.001]	.115
		Vocab	Age	−0.4	.004	.038*	[−0.003 −0.0006]	.061
	FU-BL	Mem	Age	−0.235	.011	.046*	[−0.001 −0.0001]	.046
		Fluid	Age	−0.272	.004	.013*	[−0.002 −0.0003]	.063
		Speed	Age	−0.293	.001	.007**	[−0.002 −0.0005]	.075
		Vocab	Age	−0.211	.02	.17	[−0.002 −0.0001]	.039
BEHAVIOR (WITH AUC)	BL	Mem	Age	−0.564	<0.001	<0.001***	[−0.038 −0.016]	.144
		Mem	NART	.401	<0.001	<0.001***	[.022 0.055]	.135
		Mem	Edu	.252	.002	.010*	[.03 0.125]	.068
		Fluid	Age	−0.536	<0.001	<0.001***	[−0.04 −0.02]	.138
		Fluid	NART	.485	<0.001	<0.001***	[.034 0.068]	.2
		Fluid	CT	−0.25	.003	.013*	[−3.33 −0.69]	.061
		Fluid	Edu	.224	.003	.013*	[.026 0.127]	.059
		Speed	Age	−0.811	<0.001	<0.001***	[−0.055 −0.031]	.262
		Speed	NART	.434	<0.001	<0.001***	[.027 0.062]	.157
		Vocab	AUC	.119	.038	.27	[.0001 0.002]	.031
		Vocab	NART	.686	<0.001	<0.001***	[.006 0.09]	.413
	FU	Mem	Age	−0.394	.002	.010*	[−0.032 −0.008]	.07
		Mem	NART	.556	<0.001	<0.001***	[.035 0.07]	.208
		Fluid	AUC	.237	<0.001	.002**	[.001 0.003]	.093
		Fluid	Age	−0.704	<0.001	<0.001***	[−0.05 −0.028]	.268
		Fluid	NART	.554	<0.001	<0.001***	[.042 0.073]	.286
		Speed	Age	−0.844	<0.001	<0.001***	[−0.06 −0.035]	.3
		Speed	NART	.321	.001	.001**	[.017 0.052]	.102
		Vocab	AUC	.133	.02	.17	[.0002 0.002]	.042
		Vocab	NART	.805	<0.001	<0.001***	[.075 0.106]	.512
	FU-BL	Mem	Age	−0.24	.008	.037*	[−0.016 −0.002]	.05
		Mem	NART	.535	<0.001	<0.001***	[.024 0.055]	.165
		Mem	ΔCT	.19	.025	.094†	[.302 4.74]	.039
		Fluid	ΔAUC	.277	<0.001	.003**	[.0006 0.002]	.094
		Fluid	Age	−0.415	<0.001	<0.001***	[−0.022 −0.009]	.163
		Fluid	NART	.428	<0.001	<0.001***	[.017 0.044]	.142
		Fluid	ΔWMH	−0.175	.024	.081†	[−0.384 −0.03]	.041
		Speed	Age	−0.356	<0.001	.0014**	[−0.019 −0.006]	.108
		Vocab	NART	.456	<0.001	<0.001***	[.017 0.047]	.139
		Vocab	Edu	−0.2	.047	.28	[−0.097 −0.0006]	.035

“AUC” represents the regression models where the AUC of B_0_ curves is the outcome measure. “SEG” represents regression models where functional segregation is outcome. “Behavior” represents regression models where behavioral performance is outcome. “Domain” represents the domain, or reference ability, of the outcome measure. FDR-correction using the Benjamini-Hochberg method was implemented, where all regressions belonging to a given domain were considered as part of a “family of hypotheses”; thus, separate corrections were performed per domain. Shaded gray rows indicate predictors that were significant after controlling for multiple comparisons. Asterisks indicate statistical significance at threshold levels *p*<0.05 (*), *p*<0.01 (**), and *p*<0.001(***). All predictors, as highlighted in Section 2.3.1., were included in each model.

*β* = Standardized coefficient beta; *p* = p-value (uncorrected); FDR-*p*= FDR-corrected p-value; CI= 95% confidence interval; $\eta_{\rho }^{2}=\text{partial eta-squared effect size}$.

## Data Availability

The data that support the findings of this study are available on an open source online platform (https://osf.io/dashboard). The data has been uploaded under the same title as the paper. Additionally, the main script for the TDA calculation has been made public on a github repository, along with baseline data of the connectivity matrices (https://github.com/GLArgiris/TDA_script.git).
