# Supplementary material for: Simple topological task-based functional connectivity features predict longitudinal behavioral change of fluid reasoning in the RANN cohort
Source: Neuroimage. Author manuscript; Available in PMC 2024 Apr 7. (PMC10999229; doi:10.1016/j.neuroimage.2023.120237)
Supplement: supplementary material [file NIHMS1979108-supplement-supplementary_material.docx]

**SUPPLEMENTARY MATERIAL**

| **MEMORY** | | | | | | | | | | |
| --- | --- | --- | --- | --- | --- | --- | --- | --- | --- | --- |
|  | | **Baseline (BL)** | | | **Follow-Up (FU)** | | | **FU-BL** | | |
|  | **Var** | **β** | **p** | **FDR-p** | **β** | **p** | **FDR-p** | **β** | **p** | **FDR-p** |
| **AUC** | Age | .004 | .975 | .975 | .1 | .45 | .605 | -.056 | .517 | .663 |
|  | NART | -.028 | .781 | .923 | -.089 | .377 | .554 | -.086 | .423 | .589 |
|  | WMH | -.016 | .895 | .957 | -.18 | .147 | .303 | -.167 | .051 | .159 |
|  | CT | .161 | .114 | .277 | .16 | .114 | .277 | .125 | .148 | .303 |
|  | Sex | **-.223** | **.007** | **.035*** | -.09 | .275 | .467 | -.083 | .326 | .509 |
|  | Edu | -.026 | .807 | .94 | **.215** | **.023** | **.088†** | .185 | .062 | .178 |
| **SEG** | Age | **-.727** | **<.001** | **<.001***** | **-.373** | **.004** | **.020*** | **-.235** | **.011** | **.046*** |
|  | NART | .132 | .141 | 303 | .033 | .731 | .877 | -.016 | .876 | .957 |
|  | WMH | .232 | .031 | 112 | .123 | .299 | .485 | .094 | .273 | .467 |
|  | CT | -.14 | .122 | 289 | .099 | .306 | .488 | .01 | .941 | .975 |
|  | Sex | .148 | .045 | 146 | .109 | .169 | .338 | .061 | .468 | .618 |
|  | Edu | -.031 | .710 | 865 | -.145 | .108 | .277 | -.116 | .239 | .437 |
| **BEHAVIOR** | AUC | -.132 | .060 | .178 | .014 | 0.857 | .957 | .053 | .520 | .663 |
|  | SEG | .087 | .268 | .467 | -.157 | .042 | .142 | -.1 | .241 | .437 |
|  | Age | **-.564** | **<.001** | **<.001***** | **-.394** | **.002** | **.010*** | **-.24** | **.008** | **.037*** |
|  | NART | **.401** | **<.000** | **<.001***** | **.556** | **<.001** | **<.001***** | **.535** | **<.001** | **<.001***** |
|  | WMH | .105 | .296 | .485 | -.021 | .854 | .957 | -.127 | .134 | .303 |
|  | CT | -.106 | .222 | .422 | -.014 | .884 | .957 | **.19** | **.025** | **.094†** |
|  | Sex | .037 | .605 | .749 | .047 | .527 | .663 | .075 | .363 | .544 |
|  | Edu | **.252** | **.002** | **.010*** | .015 | .860 | .957 | -.18 | .074 | .201 |

| **FLUID REASONING** | | | | | | | | | | |
| --- | --- | --- | --- | --- | --- | --- | --- | --- | --- | --- |
|  | | **Baseline (BL)** | | | **Follow-Up (FU)** | | | **FU-BL** | | |
|  | **Var** | **β** | **p** | **FDR-p** | **β** | **p** | **FDR-p** | **β** | **p** | **FDR-p** |
| **AUC** | Age | -.22 | .114 | .270 | -.125 | .347 | .520 | **-.262** | **.004** | **.016*** |
|  | NART | .074 | .459 | .618 | -.019 | .848 | .906 | .001 | .988 | .988 |
|  | WMH | .157 | .184 | .359 | -.154 | .214 | .397 | .011 | .921 | .945 |
|  | CT | .108 | .292 | .455 | .049 | .625 | .761 | .094 | .258 | .428 |
|  | Sex | -.125 | .127 | .284 | -.178 | .032 | .104 | -.143 | .094 | .233 |
|  | Edu | .1 | .278 | .452 | .189 | .044 | .122 | .207 | .037 | .112 |
| **SEG** | Age | **-.623** | **<.001** | **<.001***** | **-.391** | **.002** | **.010*** | **-.272** | **.004** | **.013*** |
|  | NART | -.024 | -0.794 | .861 | -.029 | .761 | .836 | -.071 | .498 | .658 |
|  | WMH | .225 | .041 | .119 | .103 | .376 | .540 | -.007 | .952 | .964 |
|  | CT | -.062 | .513 | .667 | .164 | .084 | .226 | -.021 | .722 | .829 |
|  | Sex | .008 | .919 | .945 | .108 | .167 | .334 | .119 | .162 | .332 |
|  | Edu | .046 | .591 | .732 | -.028 | .752 | .836 | -.011 | .916 | .945 |
| **BEHAVIOR** | AUC | .08 | .237 | .420 | **.237** | **<.001** | **.002**** | **.277** | **<.001** | **.003**** |
|  | SEG | -.077 | .289 | .455 | -.119 | .092 | .233 | -.104 | .21 | .4 |
|  | Age | **-.536** | **.001** | **<.001***** | **-.704** | **<.001** | **.001***** | **-.415** | **<.001** | **<.001***** |
|  | NART | **.485** | **.001** | **<.001***** | **.554** | **<.001** | **.001***** | **.428** | **<.001** | **<.001***** |
|  | WMH | -.071 | .460 | .618 | .137 | .146 | .316 | **-.175** | **.024** | **.081†** |
|  | **CT** | **-.25** | **.003** | **.013*** | -.124 | .095 | .233 | .027 | .689 | .815 |
|  | Sex | .077 | .247 | .421 | .052 | .404 | .563 | .045 | .558 | .702 |
|  | Edu | **.224** | **.003** | **.013*** | .063 | .374 | .540 | -.136 | .125 | .284 |

| **PROCESSING SPEED** | | | | | | | | | | |
| --- | --- | --- | --- | --- | --- | --- | --- | --- | --- | --- |
|  | | **Baseline (BL)** | | | **Follow-Up (FU)** | | | **FU-BL** | | |
|  | **Var** | **β** | **p** | **FDR-p** | **β** | **p** | **FDR-p** | **β** | **p** | **FDR-p** |
| **AUC** | Age | -.088 | .516 | .789 | -.037 | .792 | .895 | -.022 | .815 | .895 |
|  | NART | -.085 | .396 | .690 | .029 | .784 | .895 | .12 | .267 | .557 |
|  | WMH | .129 | .283 | .557 | -.027 | .835 | .895 | -.124 | .160 | .431 |
|  | CT | -.091 | .361 | .655 | -.046 | .665 | .879 | .048 | .554 | .789 |
|  | Sex | -.13 | .116 | .378 | -.084 | .327 | .608 | -.074 | .398 | .690 |
|  | Edu | .146 | .118 | .378 | .127 | .195 | .476 | .034 | .738 | .895 |
| **SEG** | Age | **-.662** | **<.001** | **<.001***** | **-.532** | **<.001** | **<.001***** | **-.293** | **.001** | **.007**** |
|  | NART | -.002 | .984 | .995 | -.003 | .975 | .995 | .045 | .659 | .879 |
|  | WMH | .131 | .202 | .477 | .078 | .497 | .789 | -.152 | .067 | .291 |
|  | CT | -.031 | .718 | .895 | .03 | .744 | .895 | .023 | .790 | .895 |
|  | Sex | -.084 | .234 | .538 | .038 | .617 | .845 | .148 | .072 | .296 |
|  | Edu | .149 | .063 | .291 | .094 | .277 | .557 | .02 | .833 | .895 |
| **BEHAVIOR** | AUC | .047 | .499 | .789 | .125 | .061 | .291 | .092 | .286 | .557 |
|  | SEG | -.004 | .960 | .995 | -.016 | .832 | .895 | .057 | .522 | .789 |
|  | Age | **-.811** | **<.001** | **<.001***** | **-.844** | **<.001** | **<.001***** | **-.356** | **<.001** | **.0014**** |
|  | NART | **.434** | **<.001** | **<.001***** | **.321** | **<.001** | **.0014**** | -.001 | .995 | .995 |
|  | WMH | .169 | .094 | .350 | .167 | .107 | .378 | .052 | .556 | .789 |
|  | CT | -.115 | .177 | .458 | -.055 | .531 | .789 | .121 | .157 | .431 |
|  | Sex | .021 | .766 | .895 | .051 | .447 | .758 | .1 | .250 | .557 |
|  | Edu | -.152 | .055 | .291 | -.105 | .182 | .458 | -.027 | .797 | .895 |

| **VOCABULARY** | | | | | | | | | | | |
| --- | --- | --- | --- | --- | --- | --- | --- | --- | --- | --- | --- |
|  | | **Baseline (BL)** | | | **Follow-Up (FU)** | | | | **FU-BL** | | |
|  | **Var** | **β** | **p** | **FDR-p** | **β** | | **p** | **FDR-p** | **β** | **p** | **FDR-p** |
| **AUC** | Age | -.109 | .443 | .818 | .077 | .589 | | .873 | -.072 | .454 | .818 |
|  | NART | -.031 | .760 | .913 | .139 | .197 | | .667 | .138 | .217 | .684 |
|  | WMH | -.008 | .950 | .950 | -.131 | .327 | | .751 | -.045 | .627 | .873 |
|  | CT | -.156 | .129 | .530 | .113 | .285 | | .751 | .03 | .795 | .913 |
|  | Sex | -.043 | .614 | .873 | -.075 | .389 | | .817 | -.052 | .569 | .873 |
|  | Edu | .093 | .326 | .751 | -.102 | .310 | | .751 | -.107 | .309 | .751 |
| **SEG** | Age | **-.575** | **<.001** | **<.001***** | **-.395** | **.004** | | **.036*** | -.212 | .022 | .170 |
|  | NART | .029 | .752 | .913 | -.016 | .876 | | .936 | .029 | .783 | .913 |
|  | WMH | .171 | .119 | .517 | .065 | .608 | | .873 | -.147 | .096 | .441 |
|  | CT | .02 | .824 | .913 | .042 | .671 | | .902 | .165 | .052 | .292 |
|  | Sex | .089 | .234 | .702 | .045 | .584 | | .873 | .04 | .644 | .881 |
|  | Edu | .088 | .294 | .751 | .083 | .376 | | .817 | .012 | .901 | .939 |
| **BEHAVIOR** | AUC | .119 | .038 | .270 | .133 | .020 | | .170 | .127 | .145 | .564 |
|  | SEG | .032 | .624 | .873 | -.016 | .800 | | .913 | -.09 | .319 | .751 |
|  | Age | .021 | .831 | .913 | .076 | .409 | | .817 | .024 | .761 | .913 |
|  | NART | **.686** | **<.001** | **<.001***** | **.805** | **<.001** | | **<.001***** | **.456** | **<.001** | **<.001***** |
|  | WMH | .062 | .459 | .818 | -.063 | .477 | | .827 | -.034 | .706 | .913 |
|  | CT | .06 | .403 | .817 | .035 | .619 | | .873 | -.118 | .175 | .651 |
|  | Sex | -.006 | .924 | .939 | -.005 | .927 | | .939 | -.024 | .783 | .913 |
|  | Edu | .08 | .219 | .684 | -.109 | .096 | | .441 | -.2 | .047 | .280 |

***ST1*. *List of all predictors for all linear regression models.*** “AUC” represents the regression models where the AUC of B_0_ curves is the outcome measure. “SEG” represents regression models where functional segregation is outcome. “Behavior” represents regression models where behavioral performance is outcome. For behavior regression models, the brain integrity and demographic predictors reported in the table are from the model including “AUC” as the brain measure. Behavioral models including “SEG” as predictor were also created; however, we only report the SEG predictor for direct comparison to AUC without brain integrity and demographic predictors. “Domain” represents the domain, or reference ability, of the outcome measure. FDR-correction using the Benjamini-Hochberg method was implemented, where all regressions belonging to a given domain were considered as part of a “family of hypotheses”; 78 predictor outcomes were thus considered per domain.

β= Standardized coefficient beta; *p*= p-value (uncorrected); FDR-p= FDR-corrected p-value.

------------


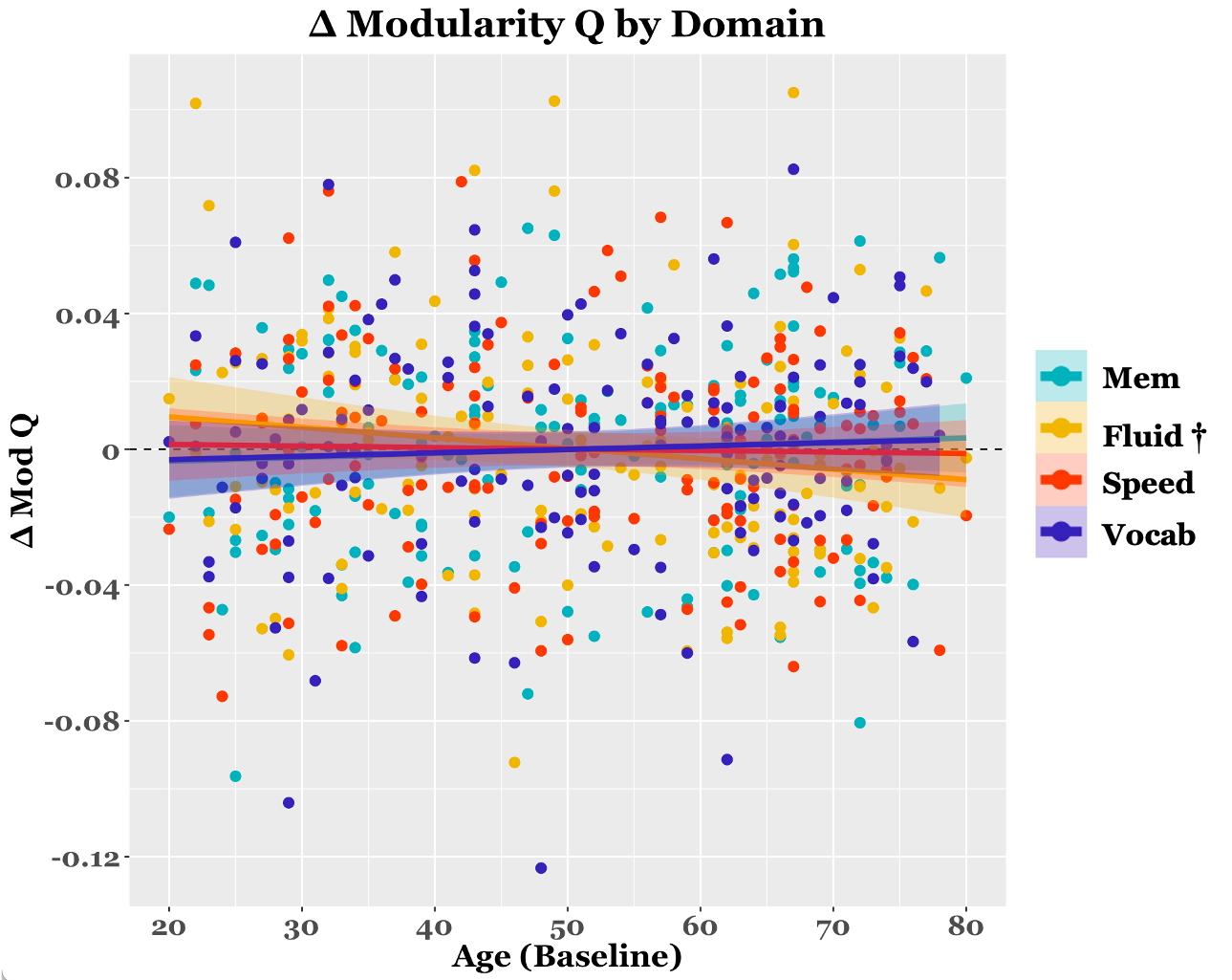


***SF1. Scatterplot depicting the relationship between baseline Age and longitudinal change in modularity Q.*** Colored dots represent each participant’s modularity Q value per domain.

Colored error ribbons represent the 95% confidence band. Change in modularity Q (i.e., ΔMod Q) values have been adjusted for baseline modularity, NART, Education, Sex, ΔCT, and ΔWMH and thus represent the raw residuals after this adjustment. A non-significant effect with p-value of 0.05<p<.1 after multiple comparisons correction was observed for the Fluid Reasoning domain.

------------

| **FLUID REASONING** | | | | | | | | | | |
| --- | --- | --- | --- | --- | --- | --- | --- | --- | --- | --- |
|  | | **Baseline (BL)** | | | **Follow-Up (FU)** | | | **FU-BL** | | |
|  | **Var** | **β** | **p** | **FDR-p** | **β** | **p** | **FDR-p** | **β** | **p** | **FDR-p** |
| **MOD Q** | Age | **-.432** | **.002** | **.010*** | -.277 | .044 | .115 | **-.214** | **.013** | **.052†** |
|  | NART | .006 | .954 | .954 | .085 | .414 | .576 | .058 | .578 | .723 |
|  | WMH | .15 | .199 | .376 | .043 | .735 | .868 | **.193** | **.026** | **.086†** |
|  | CT | -.148 | .143 | .317 | -.101 | .326 | .509 | .015 | .779 | .893 |
|  | Sex | .116 | .153 | .317 | .178 | .037 | .112 | .177 | .040 | .113 |
|  | Edu | -.016 | .860 | .934 | .052 | .593 | .723 | .122 | .202 | .376 |
| **BEHAVIOR** | MOD Q | .075 | .278 | .492 | .011 | .862 | .934 | .075 | .339 | .509 |
|  | Age | **-.518** | **<.001** | **<.001***** | **-.729** | **<.001** | **<.001***** | **-.474** | **<.001** | **<.001***** |
|  | NART | **.485** | **<.001** | **<.001***** | **.547** | **<.001** | **<.001***** | **.427** | **<.001** | **<.001***** |
|  | WMH | -.069 | .470 | .633 | .099 | .313 | .509 | **-.186** | **.024** | **.085†** |
|  | CT | **-.23** | **.007** | **.030*** | -.112 | .155 | .317 | .051 | .495 | .644 |
|  | Sex | .058 | .385 | .555 | .001 | .906 | .954 | -.005 | .941 | .954 |
|  | Edu | **.235** | **.002** | **.010*** | .108 | .143 | .317 | -.089 | .328 | .509 |

***ST2*. *List of predictors for Fluid Reasoning linear regression models of modularity Q.*** “MOD Q” represents the regression models where modularity Q is the outcome measure. “Behavior” represents regression models where behavioral performance is outcome and modularity Q is included as the brain measure in the model. Given that these analyses were supplementary to the main aim, FDR-correction using the Benjamini-Hochberg method was implemented separately for models containing the modularity Q metric. Similar to the main analyses, all regressions belonging to a given domain were considered as part of a “family of hypotheses”; in this case, 39 predictor outcomes were thus considered per domain. As can be observed from the table, modularity Q did not predict behavioral performance at any time point.

β= Standardized coefficient beta; *p*= p-value (uncorrected); FDR-p= FDR-corrected p-value.

| **FLUID REASONING** | | | | | | | | | | |
| --- | --- | --- | --- | --- | --- | --- | --- | --- | --- | --- |
|  | | **Baseline (BL)** | | | **Follow-Up (FU)** | | | **FU-BL** | | |
|  | **Var** | **β** | **p** | **FDR-p** | **β** | **p** | **FDR-p** | **β** | **p** | **FDR-p** |
| **AUC_t** | Age | -.079 | .568 | .633 | -.020 | .885 | .909 | **-.225** | **.014** | **.047*** |
|  | NART | .030 | .764 | .828 | -.111 | .280 | .433 | -.099 | .355 | .513 |
|  | WMH | .151 | .199 | .352 | -.140 | .268 | .433 | .057 | .524 | .601 |
|  | CT | .199 | .051 | .123 | .089 | .384 | .516 | .072 | .397 | .516 |
|  | Sex | -.090 | .271 | .433 | **-.198** | .**019** | **.052†** | **-.224** | **.009** | **.040*** |
|  | Edu | .127 | .172 | .334 | **.230** | .**017** | **.050†** | **.244** | **.014** | **.047*** |
| **BEHAVIOR** | AUC_t | -.004 | .950 | .950 | **.156** | **.020** | **.053†** | **.205** | **.339** | **.047*** |
|  | Age | -.575 | **<.001** | **<.001***** | **-.731** | **<.001** | **<.001***** | -.435 | **<.001** | **<.001***** |
|  | NART | .475 | **<.001** | **<.001***** | **.567** | **<.001** | **<.001***** | .430 | **<.001** | **<.001***** |
|  | WMH | -.022 | .819 | .864 | .127 | .196 | .352 | -.150 | .066 | .150 |
|  | CT | **-.264** | **.002** | **.012*** | -.128 | .107 | .220 | .061 | .424 | .533 |
|  | Sex | .044 | .508 | .6 | .050 | .447 | .545 | .086 | .289 | .433 |
|  | Edu | **.236** | **.002** | **.012*** | .066 | .378 | .516 | -.153 | .107 | .220 |

***ST3*. *List of predictors for Fluid Reasoning linear regression models of AUC calculated on task-regressed time series (AUC_t).*** Before calculating AUC, task onsets and offsets were modeled and regressed out from the time series. “AUC_t|” represents AUC calculated on this altered time series. Given that these analyses were supplementary to the main aim of the paper, FDR-correction using the Benjamini-Hochberg method was implemented separately for these models; thus, 39 predictor outcomes were thus considered per the Fluid Reasoning domain. As can be observed from the table, AUC_t did not display significant relationships with Age nor predict behavioral performance at any time point.

β= Standardized coefficient beta; *p*= p-value (uncorrected); FDR-p= FDR-corrected p-value.

| **FLUID REASONING** | | | | | | | | | | |
| --- | --- | --- | --- | --- | --- | --- | --- | --- | --- | --- |
|  | | **Baseline (BL)** | | | **Follow-Up (FU)** | | | **FU-BL** | | |
|  | **Var** | **β** | **p** | **FDR-p** | **β** | **p** | **FDR-p** | **β** | **p** | **FDR-p** |
| **AUC (r)** | Age | .106 | .444 | .668 | .110 | .406 | .668 | .141 | .115 | .299 |
|  | NART | -.074 | .462 | .668 | -.032 | .755 | .835 | -.016 | .878 | .882 |
|  | WMH | .028 | .814 | .858 | .073 | .559 | .752 | -.027 | .771 | .835 |
|  | CT | -.173 | .091 | .262 | -.069 | .491 | .684 | -.148 | .080 | .262 |
|  | Sex | .012 | .882 | .882 | .139 | .094 | .262 | .067 | .434 | .668 |
|  | Edu | .081 | .380 | .668 | -.179 | .060 | .233 | -.214 | .032 | .138 |
| **BEHAVIOR** | AUC (r) | -.032 | .639 | .803 | -.048 | .461 | .668 | -.035 | .339 | .669 |
|  | Age | -.565 | **<.001** | **<.001***** | -.728 | **<.001** | **<.001***** | -.471 | **<.001** | **<.001***** |
|  | NART | .472 | **<.001** | **<.001***** | .549 | **<.001** | **<.001***** | .424 | **<.001** | **<.001***** |
|  | WMH | -.037 | .704 | .832 | .109 | .262 | .567 | **-.146** | **.024** | **.073†** |
|  | CT | **-.259** | **.003** | **.015*** | -.106 | .172 | .418 | .067 | .380 | .668 |
|  | Sex | .052 | .436 | .555 | .033 | .612 | .796 | .024 | .769 | .835 |
|  | Edu | **.228** | **.003** | **.016*** | .093 | .208 | .478 | -.101 | .286 | .587 |

***ST4*. *List of predictors for Fluid Reasoning linear regression models of AUC with r-threshold.*** “AUC (r)” represents the regression models where AUC calculated from correlation threshold instead of edge density, is the outcome measure and is considered as the brain measure in the behavioral model. Given that these analyses were supplementary to the main aim, FDR-correction using the Benjamini-Hochberg method was implemented separately for models containing the AUC (r) metric; thus, 39 predictor outcomes were considered for the Fluid Reasoning domain. As can be observed from the table, AUC (r) did not display significant relationships with Age nor predict behavioral performance at any time point.

β= Standardized coefficient beta; *p*= p-value (uncorrected); FDR-p= FDR-corrected p-value.
